# Intracerebroventricular injection of autologous, Wnt‐activated, adipose‐derived stem cells for the treatment of Alzheimer’s Disease: Experience with the first patient of a “First in Human” Phase 1 FDA trial

**DOI:** 10.1002/alz.094645

**Published:** 2025-01-09

**Authors:** Christopher M. Duma, Hans Keirstead, Gabriel Nistor, Robert Lynn, Jessica J. Buxton, Sawyer H. Farmer, Karlyssa Chung, Ashley Harris

**Affiliations:** ^1^ Regeneration Biomedical, Inc., Newport Beach, CA USA; ^2^ University of California, Berkeley, Berkeley, CA USA; ^3^ Albany Medical College, Albany, NY USA; ^4^ Brain and Spine Surgeons of Orange County, Newport Beach, CA USA

## Abstract

**Background:**

It has been more than 20 years for a new treatment for Alzheimer’s Disease (AD) to emerge. This treatment has recently been in the form of a monoclonal antibody targeting the end‐products of neuronal death. We are testing the safety of a novel approach using activated stem cells injected directly into the ventricles of the brain.

**Method:**

After animal testing, we received FDA clearance for a 3 + 3, Phase 1 trial in 9 patients using escalating doses of the test product. The first patient was screened for age less than 80 years, FAST stage 4 or 5, having a Mini Mental Status Exam (MMSE) between 10 and 20, as well as exhibiting brain PET and CSF AD markers. The patient underwent a 3‐step process of liposuction, Ommaya reservoir implantation, and injection following cell selection and expansion based on Wnt expression in the lab (the test product). The patient was admitted to the hospital 43 days after initial lipoaspirate cell expansion and Wnt expression selection, for the injection, and observed overnight. Five ml. of the test product containing 2M cells was injected. Vital signs (VS) and clinical examinations were performed per protocol. CSF analysis, MMSE, amyloid PET and MRI imaging will be performed at regular intervals post‐injection for 1 year.

**Result:**

There were no adverse events following any of the first 2 steps in the patient’s procedures. The injection process required 3 minutes to perform, without anesthetic. This was well‐tolerated with no immediate or remote headache, nausea, vomiting or change in VS up to 5 weeks post‐injection as of this publication.

**Conclusion:**

This “first in human” trial of intracerebroventricular injection of Wnt‐expressing, adipose‐derived stem cells appears well‐tolerated and safe for our first injected patient with a minimum of 5 weeks followup. Our intention is to submit parallel Phase 2 trials to the FDA using this Phase 1 safety data, for treatment of patients with AD, MS‐P, Parkinson’s Disease, CTE, and ALS.

